# Ex vivo innate responses to particulate matter from livestock farms in asthma patients and healthy individuals

**DOI:** 10.1186/s12940-020-00632-8

**Published:** 2020-07-03

**Authors:** Linsey E. S. de Groot, Dingyu Liu, Barbara S. Dierdorp, Niki Fens, Marianne A. van de Pol, Peter J. Sterk, Wim Kulik, Miriam E. Gerlofs-Nijland, Flemming R. Cassee, Elena Pinelli, René Lutter

**Affiliations:** 1 Department of Respiratory Medicine, Amsterdam UMC, University of Amsterdam, Amsterdam, The Netherlands; 2 Department of Experimental Immunology (Amsterdam Infection & Immunity Institute), Amsterdam UMC, University of Amsterdam, Amsterdam, The Netherlands; 3 Centre for Sustainability, Environment and Health, National Institute for Public Health and the Environment, Bilthoven, The Netherlands; 4 Institute for Risk Assessment Sciences, Utrecht University, Utrecht, The Netherlands; 5 Laboratory Genetic Metabolic Diseases, Amsterdam UMC, University of Amsterdam, Amsterdam, The Netherlands; 6 Centre for Immunology of Infectious Diseases and Vaccines, National Institute for Public Health and the Environment, Bilthoven, The Netherlands

**Keywords:** Asthma, Inflammation, Oxidative stress, Particulate matter, Peripheral blood mononuclear cells

## Abstract

**Background:**

Asthma patients suffer from periodic acute worsening of symptoms (i.e. loss of asthma control or exacerbations), triggered by a variety of exogenous stimuli. With the growing awareness that air pollutants impact respiratory diseases, we investigated whether particulate matter (PM) derived from various livestock farms (BioPM) differentially affected innate and oxidative stress responses in asthma and health.

**Methods:**

Peripheral blood mononuclear cells (PBMCs), collected from patients sequentially before and during loss of asthma control and from healthy individuals, were exposed to BioPM collected from chicken, goat and pig farms (1 and 5 μg/ml), with or without pre-treatment with antioxidants. Cytokine release and oxidative stress were assessed.

**Results:**

PBMCs produced IFNγ, IL-1β, IL-10 and TNFα upon stimulation with BioPM, with that from pig farms inducing the highest cytokine levels. Overall, cytokine production was irrespective of the presence or state of disease. However, PBMCs from stable asthma patients upon exposure to the three BioPM showed more extreme TNFα responses than those from healthy subjects. Furthermore, PBMCs obtained during loss of asthma control that were exposed to BioPM from pig farms showed enhanced IFNγ release as well as decreased oxidative stress levels upon pre-treatment with N-acetylcysteine (NAC) compared to stable disease. NAC, but not superoxide dismutase and catalase, also counteracted BioPM-induced cytokine release, indicating the importance of intracellular reactive oxygen species in the production of cytokines.

**Conclusions:**

BioPM triggered enhanced pro-inflammatory responses by PBMCs from both healthy subjects and asthma patients, with those from patients during loss of asthma control showing increased susceptibility to BioPM from pig farms in particular.

## Introduction

Asthma is a chronic inflammatory lung disease associated with reversible airway obstruction and increased responsiveness of the airways to a variety of stimuli (also known as bronchial hyperresponsiveness). It is a heterogeneous disease with e.g. differences in treatment, severity and time of onset. To some extent this heterogeneity is reflected in airway inflammation, like a more eosinophilic versus a more neutrophilic inflammation, but increased levels of oxidative damage are seen in virtually all patients. Asthma patients may suffer from periodic acute worsening of symptoms, referred to as loss of asthma control or exacerbations, that can be triggered by several exogenous factors, including viruses and allergens [1].

Recently, air pollution from ozone, nitrogen dioxides and particulate matter (PM), including traffic- and livestock-related emissions, has received increasing attention as it exacerbates and even may induce asthma [2–4] and was shown to contribute to asthma mortality [5]. Traffic-related PM drives the transcription of inflammatory mediators relevant to asthma and is a potent inducer of oxidative stress [6], as many of its components may act as a source of free radicals. This is unlikely to be the case for PM collected from specific livestock farms (BioPM). BioPM, however, was shown to contain multiple Toll-like receptor (TLR) ligands and even microorganisms or parts thereof, with distinct microbiota profiles associated with corresponding animal species [7].

In this study, we evaluated whether BioPM triggers distinct innate responses by peripheral blood mononuclear cells (PBMCs) from clinically stable asthma patients as compared to healthy controls. As airway inflammation worsens during loss of asthma control, we have also collected PBMCs from those patients of whom stable samples were obtained, but now during corticosteroid withdrawal-induced loss of asthma control. This allowed us to determine whether the innate responses to BioPM in asthma were modulated compared to baseline. BioPM derived from chicken, goat and pig farms, which are considered major sources of BioPM in The Netherlands, were compared. In addition, we aimed to clarify whether BioPM exerts its effects via oxidative stress-dependent mechanisms.

## Methods

### BioPM sampling period, sites and procedure

Ambient fine (< 2.5 μm, Mass Medium Aerodynamic Diameter) PM was collected at three livestock farms in The Netherlands from July 2016 to July 2017, including one chicken, one goat and one pig farm, all located in the central region of The Netherlands. Per site, sampling was carried out for two to 6 days and for 6 hours per day (between 09:00 and 16:00 h) in order to collect sufficient material. The daily collected BioPM from each site was pooled in order to carry out the current study. Characteristic features of the collected BioPM for each site and detailed description of the sampling dates and procedures during the sampling collection is described elsewhere [7]. All BioPM were collected in demineralized water using a Versatile Aerosol Concentration Enrichment System as described previously [8].

### Subjects

Patients with mild to moderate allergic asthma originated from a standardized prospective inhaled corticosteroid (ICS) interruption study [9–11]. All were current non-smokers, treated with a stable dose of ICS (≥500 μg fluticasone or equivalent) and no systemic steroids, anti-immunoglobulin E (IgE) or antibiotic therapy. The study design included a baseline visit and a loss of disease control visit. Following baseline measurements, patients were instructed to abruptly discontinue the use of ICS until loss of asthma control occurred (or for a maximum of 8 weeks), which was defined as meeting two out of the three criteria mentioned below. Then, the second visit was scheduled. Criteria for loss of asthma control included: (1) morning peak expiratory flow < 80% of baseline on at least two consecutive days, (2) wakening due to asthma on at least two consecutive nights and (3) use of more than eight puffs short-acting β_2_-agonist per day on at least two consecutive days. The study was approved by the AMC Medical Ethics Committee (2011_082#B201152) and registered at the Netherlands Trial Register (NTR3316). All participants provided written informed consent. Healthy controls were recruited in accordance with a study protocol that was reviewed by the AMC Medical Ethics Committee (2015_074). The need for ethical approval was waived. Prior to sample donation, all donors gave informed consent. In the present study, we compared 10 asthma patients at stable disease and during loss of control and 10 healthy volunteers.

### Processing and analysis of blood

Venous blood was collected in serum and heparin tubes. Total IgE in serum was determined by ImmunoCAP (Phadia AB, Uppsala, Sweden). PBMCs were isolated from heparin blood using standard density gradient techniques and stored in liquid nitrogen until further analysis.

### PBMC stimulation

For stimulations, PBMCs were thawed, washed, counted on the Coulter counter (Beckman Coulter, Brea, CA, USA) and diluted to 10^6^/ml culture medium. The optimal BioPM concentration (range: 0.01 to 50 μg/ml) was determined based on cell viability and cytokine production. For experiments described here, PBMCs were plated in the presence or absence of BioPM collected from chicken, goat or pig farms (1 or 5 μg/ml), with or without 1 h pre-treatment with N-acetylcysteine (NAC) (Sigma-Aldrich, Saint Louis, MO, USA; 1 or 10 mM) or a combination of superoxide dismutase (SOD) (Sigma-Aldrich; 100 μg/ml) and catalase (Boehringer Mannheim GmbH, Mannheim, Germany; 50 μg/ml) and incubated for 20 h. Supernatant was collected for subsequent assays.

### Viability

To assess the potential cytotoxic effect of BioPM and NAC, PBMC viability was determined using Cell Proliferation Reagent WST-1 (Roche Diagnostics GmbH, Mannheim, Germany) according to the instructions of the manufacturer.

### Luminex

Interferon (IFN) γ, interleukin (IL)-10, IL-1β and tumor necrosis factor (TNF) α were measured using R&D Systems (Minneapolis, MN, USA) reagents according to the instructions of the manufacturer and read on a Bioplex 200 (Bio-Rad, Hercules, CA, USA). Possible interference of NAC with Luminex antibodies was excluded by direct addition of 10 mM NAC to the standards.

### Mass spectrometry

Malondialdehyde (MDA) was determined by ultra-performance liquid chromatography-tandem mass spectrometry as described previously [12].

### Statistical analysis

Statistical analysis was performed using GraphPad Prism 8.0 (GraphPad Software, La Jolla, CA, USA). Data are presented as mean ± SEM and were analyzed using paired or unpaired t-tests, F-tests to compare variances, RM one-way ANOVA or mixed-effects analysis where appropriate. *P*-values < 0.05 were considered statistically significant.

## Results

Asthma patients were on average 28.3 ± 2.9 years of age and 70% of them was female. Clinical characteristics at stable disease and during loss of control are summarized in Table 1. The average time until loss of asthma control was 31.5 ± 4.8 days and this was associated with a significant increase in Asthma Control Questionnaire and Wisconsin Upper Respiratory Symptom Survey scores and a significant decrease in forced expiratory volume in 1 second (FEV_1_) % predicted compared to baseline. Furthermore, loss of asthma control was accompanied by increased sputum eosinophils, whereas neutrophils remained unaffected. For the healthy controls, 30% had total IgE levels over 100 kU/L and were considered allergic. Monocyte percentages in thawed PBMCs did not differ between asthma patients and healthy volunteers (14.87 ± 1.22 versus 12.37 ± 0.85; *p* = 0.11) and were not affected by loss of asthma control (14.38 ± 0.83; *p* = 0.51 versus stable asthma).

**Table 1:** Clinical characteristics of asthma patients at stable disease and during loss of control

	Stable	Loss of control	***P***-value
ACQ	6.30 ± 1.04	20.60 ± 0.88	**< 0.0001**
WURSS	41.10 ± 8.97	60.10 ± 10.48	**0.04**
FEV_1_% predicted	103.0 ± 3.70	90.30 ± 5.89	**0.01**
FeNO (ppb)	39.40 ± 13.33	61.70 ± 15.40	0.24
Sputum eosinophils (%)	2.58 ± 1.45	13.46 ± 4.62	**0.03**
Sputum neutrophils (%)	41.90 ± 12.20	44.56 ± 8.30	0.65
Blood eosinophils (%)	3.39 ± 0.85	6.33 ± 2.15	0.07
Blood eosinophils (10^9^/L)	0.22 ± 0.06	0.43 ± 0.17	0.13
Blood neutrophils (%)	55.19 ± 3.58	52.86 ± 3.23	0.19
Blood neutrophils (10^9^/L)	3.80 ± 0.72	3.34 ± 0.32	0.35

Data (mean ± SEM) for the 10 asthma patients of whom PBMCs were used. Data for all patients included in the corticosteroid interruption study is provided elsewhere [9–11]. ACQ, Asthma Control Questionnaire; WURSS, Wisconsin Upper Respiratory Symptom Survey; FEV_1_, forced expiratory volume in 1 second; FeNO, fraction exhaled nitric oxide

**Fig. 1:**
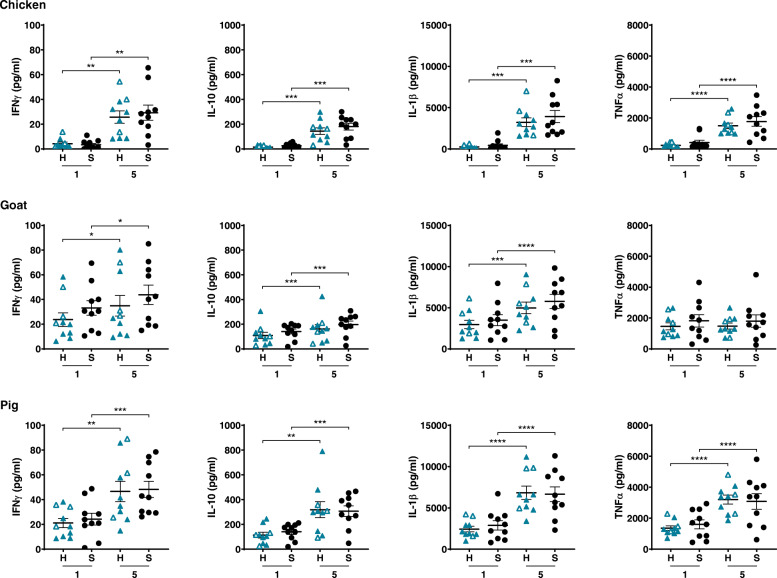
Cytokine production induced by BioPM (1 or 5 μg/ml) collected from chicken, goat and pig farms in PBMCs from healthy volunteers (H; blue triangles; closed, non-allergic; open, allergic) and stable asthma patients (S; black dots); *n* = 10. *p < 0.05, **p < 0.01, ***p < 0.001, ****p < 0.0001

For all three groups, no cytokine levels were detected when PBMCs were cultured in medium only (not shown). One and 5 μg/ml BioPM collected from chicken, goat and pig farms induced the production of IFNγ, IL-10, IL-1β and TNFα by PBMCs from stable asthma patients and healthy volunteers in an apparent concentration-dependent manner (except for TNFα production induced by BioPM from the goat farm) (Fig. 1). Cell viability remained > 80% (not shown). The source of BioPM determined the magnitude of cytokine production, with exposure to the pig farm generally inducing the highest cytokine production and exposure to the chicken farm the lowest. The response to BioPM in healthy controls was not associated with allergic status, suggesting that the results for asthma patients are not allergy-related. No significant differences in response were detected between stable asthma patients and healthy controls. Yet, PBMCs from the asthma group compared to those from healthy controls showed more variability with, for the same patients, extreme (high and low) TNFα responses to the three BioPM (*p* < 0.01, *p* = 0.11 and *p* = 0.07 for 1 μg/ml and *p* = 0.13, *p* < 0.05 and *p* = 0.12 for 5 μg/ml BioPM from chicken, goat and pig farms, respectively).

**Fig. 2:**
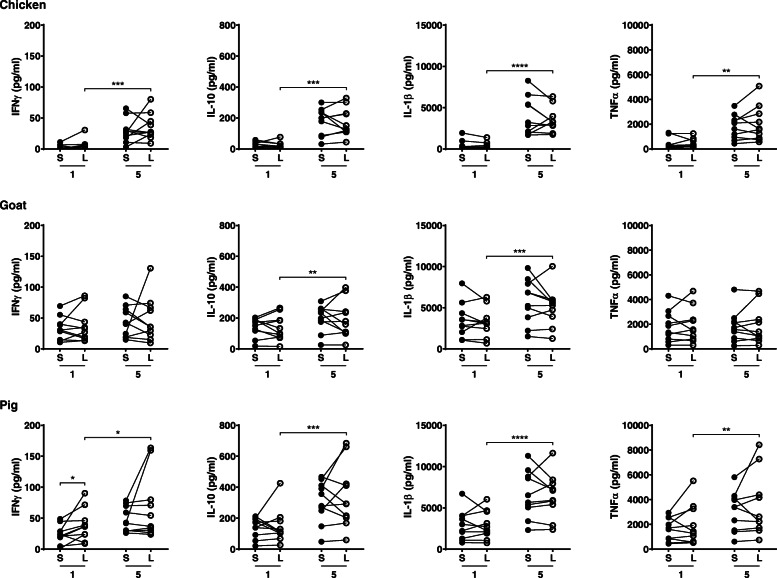
Cytokine production induced by BioPM (1 or 5 μg/ml) collected from chicken, goat and pig farms in PBMCs from asthma patients at stable disease (S; closed dots) and during loss of control (L; open dots); *n* = 10. *p < 0.05, **p < 0.01, ***p < 0.001, ****p < 0.0001. Significance for stable disease 1 versus 5 μg/ml is not shown

We then questioned whether PBMCs would respond differently when obtained from the same patients experiencing loss of asthma control compared to stable disease. Again, BioPM induced cytokine production in a concentration-dependent manner by PBMCs from patients during loss of asthma control (with the exception of IFNγ and TNFα production induced by BioPM from the goat farm) (Fig. 2). PBMCs from asthma patients during loss of control showed enhanced IFNγ levels compared to stable disease after exposure to 1 μg/ml BioPM collected from the pig farm, but no other differences in BioPM-induced cytokine production were found between the two groups.

**Fig. 3:**
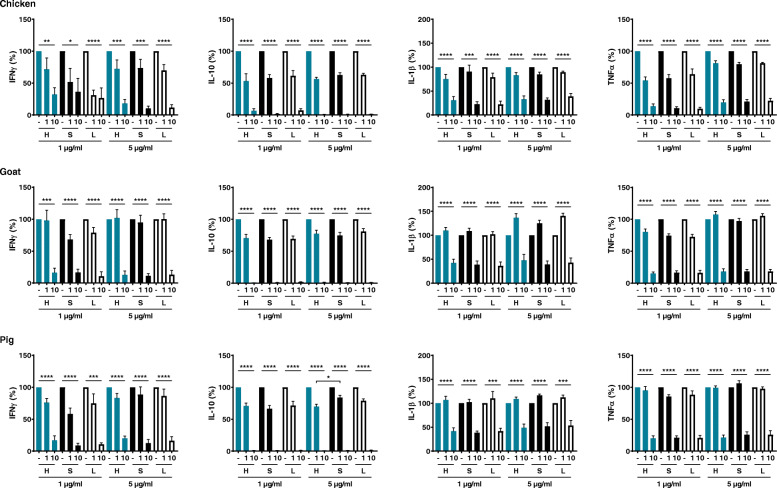
Effect of pre-treatment with NAC (1 or 10 mM) on cytokine production induced by BioPM (1 or 5 μg/ml) collected from chicken, goat and pig farms in PBMCs from healthy volunteers (H; blue) and asthma patients at stable disease (S; black) and during loss of control (L; white); *n* = 10. *p < 0.05, **p < 0.01, ***p < 0.001, ****p < 0.0001

Pre-treatment of PBMCs with the antioxidant NAC attenuated cytokine production induced by BioPM from all three farms in all three groups (Fig. 3). NAC at a concentration of 10 mM was generally more efficient than 1 mM, with 1 mM in some cases not having any effect or even resulting in a minor increase in cytokine production. NAC by itself did not induce cytokine release, nor did it affect cell viability (not shown). The effect of NAC appeared to be most pronounced for IL-10, where cytokine production was almost completely abolished when PBMCs were pre-treated with 10 mM NAC. In general, again no differences between healthy controls and asthma patients during stable disease and during loss of control were found, although 5 μg/ml pig farm BioPM-exposed PBMCs from asthma patients were less responsive to pre-treatment with 1 mM NAC in terms of IL-10 production. The effect of NAC was, especially at 1 mM, most pronounced in the chicken farm, which inversely parallels the magnitude of cytokine production.

**Fig. 4:**
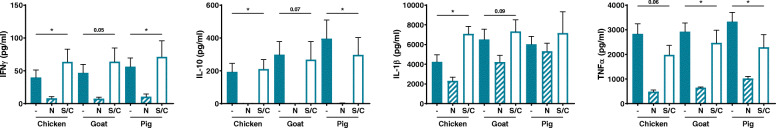
Effect of pre-treatment with NAC (N; striped; 10 mM) or SOD combined with catalase (S/C; white; 100 and 50 μg/ml, respectively) on cytokine production induced by BioPM (5 μg/ml) collected from chicken, goat and pig farms without pre-treatment (blue) in PBMCs from healthy volunteers; *n* = 4. *p < 0.05

In order to discriminate between intracellular (e.g. mitochondrial) and extracellular reactive oxygen species (ROS), we used the antioxidant combination of SOD and catalase, both large molecules that will not directly enter cells. In contrast to NAC, the combination of SOD and catalase did not inhibit BioPM-induced release of cytokines (Fig. 4). In fact, the combination of SOD and catalase, without additional exposure to BioPM, resulted in enhanced cytokine production compared to unstimulated PBMCs (not shown). Why SOD and catalase induced cytokine production by itself remains unclear, but since both commercially acquired enzymes were purified from tissues this increase could possibly be related to contaminants.

**Fig. 5:**
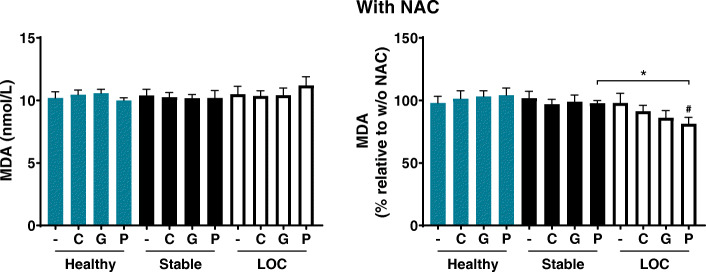
Effect of BioPM (5 μg/ml) collected from chicken (C), goat (G) and pig (P) farms and pre-treatment with NAC (10 mM) on oxidative stress levels in PBMCs from healthy volunteers (blue) and asthma patients at stable disease (black) and during loss of control (LOC; white); *n* = 5. *p < 0.05, ^#^*p* < 0.05 compared to no pre-treatment (100%)

Oxidative stress assessed by the lipid peroxidation product MDA in culture medium from PBMCs was not affected by exposure to BioPM and no differences were detected between groups or farms, although pig farm BioPM tended to slightly increase MDA levels in the loss of asthma control group (Fig. 5). Pre-treatment with 10 mM NAC was able to lower levels of MDA in PBMCs from asthma patients during loss of control after exposure to BioPM collected from pig farms, but not in PBMCs from any of the other groups or after exposure to BioPM collected from chicken or goat farms. This was also the only condition being significantly lower compared to the stable disease counterparts.

## Discussion

There is increasing evidence that PM from livestock affects respiratory diseases including asthma [13–15]. Whereas traffic-related PM effects are claimed to be mediated by ROS due to the presence of carbon or quinone species, this is less likely for BioPM. In the present study, we demonstrated that BioPM derived from chicken, goat and pig farms induced cytokine production by PBMCs from healthy and asthmatic individuals (potency rank: pig>goat> > chicken) with an apparent dose-dependency. These inflammatory events were abrogated by pre-treatment with NAC, but not SOD and catalase, suggestive of a mechanism (partly) related to intracellular ROS induced by activation of TLRs. No marked differences in inflammatory response to BioPM were detected between PBMCs from healthy controls and asthma patients. However, PBMCs obtained during loss of asthma control demonstrated an enhanced IFNγ response upon exposure to BioPM from the pig farm, which was paralleled by oxidative stress.

In the current study we focused on mediators generated particularly by monocytes/macrophages as initial drivers of the inflammatory response to BioPM. This does not exclude that BioPM may also trigger e.g. T helper 2 (Th2) responses, although in our pilot studies we found no production of IL-4, IL-5 and IL-13 by PBMCs after 20 h of exposure to BioPM (not shown). We did measure increased levels of IFNγ, IL-10, IL-1β and TNFα, which parallels earlier findings where levels of IL-1β in PBMCs and TNFα in serum from healthy volunteers were significantly higher after exposure to dust from swine confinement buildings [16], indicating that these cytokines may be associated with regulating the inflammatory response after inhalation of BioPM. Furthermore, work with human U937 macrophages showed increased TNFα mRNA levels and other pro-inflammatory marker genes after exposure to PM collected from dairy farms [17]. The large quantities of IL-1β produced in the present study are suggestive of the involvement of inflammasomes, which have been linked to asthma severity and steroid resistance [18–20]. TNFα is also of interest in the context of asthma as it was reported to determine the severity of hyperresponsiveness [21] and contribute to an exaggerated inflammatory response by bronchial epithelial cells from most asthma patients in the presence of IL-17 [22]. Here, the BioPM-induced TNFα response tended to be more variable with some extremes (high and low) for PBMCs from asthma patients compared to those from healthy subjects, although no differences were observed when comparing the asthma population as a whole. Nevertheless, as this extreme TNFα production was found for the same patients with all three BioPM and not for the three other cytokines, this indicates that this is a genuine finding. This also fits the concept that asthma is a heterogeneous disease, indicating that some asthma phenotypes may experience more severe inflammatory events in response to BioPM. We did not find any other differences in cytokine production between stable asthma patients and healthy volunteers, which is in agreement with blood responsiveness upon ex vivo stimulation with EHC-93 urban dust [23] and inflammatory responses upon in vivo exposure to ambient particles collected in Los Angeles [24]. On the other hand, it contradicts several other studies that found attenuated responses to diesel exhaust in asthmatic individuals compared to healthy controls [25, 26], which may relate to the specific nature of this PM. Similarly, levels of the oxidative stress marker MDA were comparable between stable asthma patients and healthy controls, yet not affected by exposure to BioPM. Previous studies, however, have shown that high pollution/PM induced oxidative stress levels in exhaled breath condensate and urine in healthy young adults and schoolchildren [27, 28], although this increase in oxidative stress was similar between non-asthmatics and asthmatics [29].

The lack of exaggerated inflammatory and oxidative stress events in PBMCs from asthma patients as a whole to BioPM was surprising as subjects with pre-existing respiratory disease are more susceptible to traffic-related PM-induced injury. This discrepancy between traffic-related PM and BioPM is presumably due to the presence of free radicals and/or chemical components in the first. The microbiota profiles present in our BioPM were identified previously, where it was also demonstrated that blocking of TLR4 interfered with cytokine production by MM6 cells stimulated with BioPM [7]. It is thus likely that mainly liposaccharides are responsible for the observed BioPM-induced inflammatory responses, although future studies should determine whether compounds that may lead to oxidative stress are truly absent in our BioPM. Clinical consequences may therefore depend on the source of PM, that may not only differ in composition but may, dependent on size, also deposit at different regions in the lung. Moreover, even though monocytes are precursors of lung macrophages, the PBMC responses examined in the current study do not necessarily reflect the contribution of macrophages to airway inflammation. As differences with local macrophages may exist, the actual damage by BioPM to the lungs from healthy individuals and asthma patients remains to be elucidated.

To the best of our knowledge, this is the first study that includes ex vivo BioPM exposure during loss of asthma control. The majority of BioPM-induced inflammatory responses was similar to that in stable asthma. We did, however, detect increased IFNγ levels during loss of asthma control after 1 but not after 5 μg/ml pig farm exposure, which suggests a lower threshold for IFNγ induction by PBMCs from these patients. Enhanced IFNγ production by PBMCs was also seen for asthmatic children, but despite similar seroprevalence not in non-asthmatic children, in an in vitro response to *Chlamydia pneumoniae*, which has been associated with asthma exacerbations [30]. Still, the pathophysiological effect of enhanced IFNγ responses is unknown, although we have shown before that the IFN response during rhinovirus-induced loss of asthma control correlates with eosinophilic inflammation and drop in FEV_1_ [31].

Pre-treatment with the antioxidant NAC suppressed BioPM-induced cytokine production in all groups and counteracted oxidative stress in pig farm BioPM-exposed PBMCs from patients during loss of asthma control. These effects are therefore probably related to defective antioxidant defenses as suggested previously in mice exposed to wildfire PM [32]. Induction of antioxidant defenses using vitamin supplementation has previously been demonstrated to attenuate the impact of air pollutants in children with asthma [33] and in mouse models of ovalbumin-induced experimental asthma [34, 35]. The complete inhibition of IL-10 release by pre-treatment with 10 mM NAC was remarkable. It is not unlikely that the production of IL-10 is more sensitive to oxidative stress than that of other cytokines measured, as was also reported before [36]. The combination of SOD and catalase did not abolish cytokine release, indicating that the induction of cytokines is mainly dependent on intracellular ROS, though validation in a larger cohort is necessary. This finding would also to a certain extent exclude the presence of a free radical source in BioPM and further supports BioPM-induced cell activation by TLR ligands and dysfunctional or increased mitochondrial respiration, leading to excessive ROS production and inflammatory events. The association between PM exposure and damaged mitochondria has been described before in healthy subjects [37].

Although PBMCs released cytokines upon exposure to BioPM from the three sources, there were some differences in response. Besides variation in potency, it became clear that BioPM from pig farms uniquely induced IFNγ production by PBMCs collected during loss of asthma control. Furthermore, this was the only BioPM where treatment with NAC counteracted oxidative stress in PBMCs from these patients. There may be several explanations for this, including size, (bio)chemical and microbial composition of pig farm-derived BioPM. As size and endotoxin levels were previously excluded as significant determinants, variation in response is possibly linked to the microbial or fungal diversity of BioPM from different farms as described by us before [7, 38]. In our study we also showed that BioPM primarily contains ligands for TLR2 and TLR4 [7]. Additional TLR5 ligands were specific for pig farms only, which may account for the present observations [39].

One of the strengths of this study is that we compared PBMCs from the same asthmatic patients during stable disease and loss of control, which allowed us to directly determine any effects induced by acute worsening of the disease. However, we do realize that this study also has several limitations, including the use of PBMCs instead of purified monocytes, monocyte-derived macrophages or airway macrophages. Whereas airway macrophages, as an important target for (Bio)PM, would have been the preferred cells of choice for this study, their collection before and during loss of control was not contemplated due to ethical constraints. Despite the potential difficulty in translating the findings to local effects, an advantage would be that ICS predominantly affect local cells and thus lung macrophages. Circulating monocytes would be less modulated, thereby limiting the possibility that the use of ICS may have affected our results. The use of PBMCs over monocyte-derived macrophages in this study was based on the potential loss of imprinting by in vitro maturation of monocytes towards macrophages and the potential loss of monocytes during their purification. Furthermore, PBMCs demonstrated differential gene expression upon diesel exhaust inhalation in healthy volunteers, including inflammatory and oxidative stress pathways [40, 41], indicating that PM effects are not limited to the airways but can also be found in the circulation. In fact, it has been suggested previously that fine PM elicits systemic effects rather than respiratory symptoms [42, 43]. Our findings are also based on relatively small sample sizes and a predominance of female subjects, who typically have more severe disease and more pronounced Th2 responses [44], which may have biased the results.

## Conclusions

In summary, BioPM induced cytokine release by PBMCs from healthy and asthmatic subjects via a mechanism partly related to ROS that is mainly generated intracellularly. At large, we found no significant differences between the responses of PBMCs from healthy controls and asthma patients, although for asthma all three BioPM induced extreme TNFα responses in the same patients. Therefore, some patients may respond in an exaggerated manner to BioPM, which likely contributes to enhanced inflammation and possibly may lead to enhanced loss of asthma control. Interestingly, PBMCs from patients during loss of asthma control showed enhanced IFNγ and oxidative stress responses upon stimulation with BioPM from the pig farm, indicating increased susceptibility to this particular livestock. These observations may become even more apparent with accumulation over time, in extreme asthma phenotypes and with other triggers of loss of control or exacerbations, with presumably major consequences for the course of asthma. Future research should include BioPM from a larger number of different animal farms and focus on the actual components responsible for inflammation and oxidative stress. Our findings also support that individuals should be made aware of the potential effect when working in or living near animal industries.

## Data Availability

All relevant data generated or analyzed during this study are included in this manuscript.

## Abbreviations

FEV_1_: Forced expiratory volume in one second
ICS: Inhaled corticosteroids
IFN: Interferon
IgE: Immunoglobulin E
IL: Interleukin
MDA: Malondialdehyde
NAC: N-acetylcysteine
PBMC: Peripheral blood mononuclear cell
PM: Particulate matter
ROS: Reactive oxygen species
SOD: Superoxide dismutase
Th2: T helper 2
TLR: Toll-like receptor
TNF: Tumor necrosis factor
